# More than a game: the impact of decent work on work engagement among athletes through the serial mediation of visions about future and sport anxiety

**DOI:** 10.3389/fpsyg.2025.1702929

**Published:** 2025-11-06

**Authors:** Mehmet Ali Horozoğlu, Ozan Korkmaz

**Affiliations:** 1 Vocational School of Social Sciences, Karamanoğlu Mehmetbey University, Karaman, Türkiye; 2 Faculty of Education, Karamanoğlu Mehmetbey University, Karaman, Türkiye

**Keywords:** decent work, work engagement, visions about future, hope, optimism, pessimism, sport anxiety

## Abstract

**Objective:**

This current study examined the serial mediating role of visions about future (hope, optimism, pessimism) and sport anxiety in the relationship between decent work and work engagement among professional athletes.

**Methods:**

The participants were 296 professional athletes (30.1% female, 69.9% male) aged between 18 and 48, actively competing in football, volleyball, or basketball leagues in Türkiye. Participants completed the Decent Work Scale, Visions About Future Scale, Sport Anxiety Scale, and Utrecht Work Engagement Scale. Structural equation modeling and bootstrapping methods were used for data analysis.

**Results:**

The results revealed that decent work positively predicted hope, optimism, and work engagement, and negatively predicted pessimism. Optimism negatively predicted sport anxiety, while pessimism positively predicted it. Sport anxiety had a significant negative effect on work engagement. Furthermore, optimism, pessimism, and sport anxiety significantly mediated the relationship between decent work and work engagement in a serial manner. Hope did not significantly mediate this relationship.

**Conclusion:**

These findings highlight the critical role of professional athletes’ future-oriented cognitions and anxiety levels in translating perceptions of decent work into higher engagement in sport. The study provides valuable insights for psychosocial interventions and organizational policies to enhance athletes’ sustainable careers.

## 1 Introduction

Today, professional athletics has evolved from being merely a performance-based endeavor into a professional practice in which individuals participate full-time. However, this transformation brings with it many structural problems specific to working life. Fundamental employment rights, such as income security, employment continuity and social protection, vary considerably in the sports industry (Ryba et al., 2015). Athletes’ workloads include intense demands not only on a physical level but also on a mental and emotional level; this process is often shaped by long working hours, low predictability and performance-based contracts (Schinke et al., 2017). The relatively short career span of professional athletes, the close dependence of livelihoods on success, and the uncertainty surrounding life after sports make professional sports a fragile profession.

At this point, the concept of decent work, which focuses on sustainability, a rights-based approach and individual well-being in working life, stands out as a strategic necessity that should also be addressed in the context of sports. The Decent work approach aims to provide a work environment where employees benefit from social security, work in safe and respectful conditions, and have access to development opportunities (International Labour Organization [ILO], 1999). This concept is critical not only for improving the working conditions of professional athletes but also for supporting their quality of life after their sport career field. The common point that studies point out is that professional athletes’ deprivation of social security increases the risk of psychological pressure and burnout, and in the long term, leads to adaptation problems in life after sports (Park et al., 2013). Therefore, adopting decent work principles among professional athletes will both strengthen the ethical infrastructure of sports and contribute to the protection of their individual well-being.

These structural vulnerabilities not only threaten athletes’ long-term well-being but also undermine their engagement in daily sport practices. Work engagement, another fundamental component of business life, is of particular importance in professions that require a high sense of belonging, such as athletics. Work engagement is defined as a multidimensional concept that reflects an individual’s sense of meaning toward their work, motivation, and commitment to the organization. It plays a decisive role in employees’ productivity, psychological resilience, and level of job satisfaction (Schaufeli et al., 2002). Work engagement in athletics is essential not only for achieving performance goals but also for coping with factors such as stress, pressure, injury risk, and career termination. Research indicates that professional athletes with high levels of work engagement experience lower burnout, report higher life satisfaction, and demonstrate greater long-term career sustainability (Lonsdale et al., 2007). In this context, the development of structural and psychosocial conditions that enhance work engagement among professional athletes can directly influence not only their performance but also their quality of life.

### 1.1 Decent work

The concept of Decent Work was first defined by the International Labour Organization [ILO] (1999) through four strategic objectives: employment, social protection, fundamental rights at work, and social dialog (Ghai, 2003; ILO, 2008). This approach offers a holistic framework that encompasses not only economic benefits but also decent conditions for individuals in their working lives. The Psychology of Working Theory (PWT), proposed by Blustein et al. (2016), reconceptualizes this notion at the individual level, defining it across five core dimensions that encompass personal needs such as physical and psychological security, adequate income, work–family balance, and access to health care (Duffy et al., 2016). In this context, decent work represents a contemporary approach to work that goes beyond the technical conditions of employment, addressing psychosocial needs such as meaning-making, self-actualization, and societal contribution.

In contemporary scholarship, the notion of decent work occupies a central place in the growing theoretical and empirical discussions within interdisciplinary domains, including psychology, sociology, and economics. Blustein et al. (2023) highlight that decent work addresses individuals’ fundamental needs in their working lives while simultaneously constituting a prerequisite for experiencing meaningful work. This underscores that decent work should be regarded not merely as an economic necessity but also as a psychological requirement. Moreover, structural factors such as income inequality, unemployment, and marginalization restrict individuals’ access to decent work, potentially leading to social exclusion and mental health problems (Benach et al., 2014; Standing, 2011). Thus, decent work constitutes a crucial concept at the nexus of social justice and individual well-being.

### 1.2 Work engagement

In recent years, work engagement—referring to employees’ dedication and motivation toward their work—has emerged as a significant focus of inquiry within organizational psychology. Employees’ work engagement is a factor that directly influences not only their individual performance but also organizational success. Within this framework, work engagement refers to a set of psychological states characterized by employees’ passion, dedication, and absorption in their work. For instance, Blomme et al. (2015) conceptualize work engagement as a state characterized by employees’ vigor, dedication, and full absorption in their work.

Work engagement is also closely associated with employees’ workplace happiness and overall life satisfaction (Ashfaq and Abid, 2022). A study highlights the positive effect of subjective well-being on work engagement. While subjective well-being reflects individuals’ overall level of life satisfaction, work engagement can be regarded as the manifestation of this satisfaction within the workplace (Garg and Singh, 2019). Thus, work engagement can be understood as a dynamic construct associated not only with performance at work but also with individuals’ overall quality of life. Furthermore, the impact of work engagement on organizational commitment and performance is of considerable significance. Research has investigated the positive impact of work engagement on teachers’ subjective well-being, revealing that work engagement, together with job satisfaction and organizational commitment, contributes to greater overall life satisfaction among teachers (Eryılmaz et al., 2021). Therefore, it can be stated that the concept of work engagement has a significant impact at both the individual and organizational levels.

### 1.3 Visions about future

Visions about future (VAF) is a fundamental psychological resource that reflects an individual’s overall perspective on the future and encompasses the dimensions of hope, optimism, and pessimism. This construct constitutes the foundation of individuals’ positive future orientations and is predominantly discussed in the literature within the contexts of positive psychology and career development (Ginevra et al., 2017). The concept of VAF was first conceptualized by Ginevra et al. (2017), and subsequent research has demonstrated its validity and reliability, including during early adolescence (Ginevra et al., 2020). The components of visions about the future, hope, optimism, and pessimism, shape individuals’ attitudes toward the future. In particular, possessing a strong future vision (characterized by high levels of hope and optimism and low levels of pessimism) is associated with the development of a positive future orientation (Korkmaz and Doğanülkü, 2022). Therefore, visions about the future are regarded as a fundamental psychological resource that supports individuals in navigating the uncertainties of their life journey (Hirschi, 2014).

The dimensions that constitute visions about the future have been shown to significantly influence individuals’ positive or negative attitudes and behaviors toward the future. Individuals with higher levels of hope and optimism tend to adopt a more positive outlook on the future and take more proactive steps toward achieving their goals (Ginevra et al., 2017; Hirschi, 2014). For instance, the career development literature has demonstrated that individuals with higher levels of optimism achieve more favorable outcomes in their professional lives and exert greater effort when faced with obstacles (Akça et al., 2018). Hirschi (2014) demonstrated that hope is a critical psychological resource for individuals to actively manage their careers and that higher levels of hope support proactive career behaviors. Similarly, Snyder (2000) emphasized that hopeful thinking provides individuals with additional motivation to overcome obstacles and to generate alternative pathways. Through the combined effects of these dimensions, optimistic and hopeful individuals tend to display greater willingness and resilience in making plans for the future, setting goals, and pursuing those goals. Research has shown that high levels of optimism and hope, together with low levels of pessimism, support the development of a positive future orientation. In contrast, a pessimistic outlook may lead individuals to experience hopelessness and anxiety about the future, thereby reducing their motivation to take initiative (Korkmaz and Doğanülkü, 2022).

In conclusion, hope and optimism, as core components visions about future, support individuals in adopting a positive outlook on the future and engaging in active efforts, whereas pessimism is regarded as a risk factor that can undermine these positive attitudes. Therefore, strengthening the positive elements of individuals’ visions about future (hope and optimism) while keeping the negative element, pessimism, at a manageable level may contribute to fostering more adaptive and constructive attitudes and behaviors toward the future (Hirschi, 2014; Seligman, 1990).

### 1.4 Sport anxiety

Sport anxiety is defined as a state of intense worry and tension that arises when athletes perceive challenging competition conditions as threatening (Martens et al., 1990). This form of anxiety may manifest either as trait anxiety, which is considered a stable personality characteristic, or as state anxiety, which is triggered by specific situations (Spielberger et al., 1983). Sport anxiety is also examined across two fundamental dimensions: cognitive anxiety and somatic anxiety. While cognitive anxiety refers to negative thoughts and worry, somatic anxiety manifests through physiological symptoms such as sweating and increased heart rate (Craft et al., 2003). Professional athletes often experience such forms of anxiety due to the pressure created by high levels of competition, and sport anxiety is regarded as a critical psychological variable influencing performance (Hanton et al., 2005).

While an optimal level of arousal may enhance athletic performance, excessive anxiety can distract athletes and significantly impair their performance (Jones and Hardy, 1990). This relationship is also explained by Yerkes and Dodson (1908) law: both very low and very high levels of arousal hinder the emergence of optimal performance. A comprehensive meta-analysis conducted by Woodman and Hardy (2003) revealed an overall negative relationship between sport anxiety and performance. In particular, under high levels of anxiety, athletes’ fear of making mistakes increases, which may trigger the phenomenon of ‘choking,’ characterized by sudden drops in performance (Mesagno and Beckmann, 2017). In another study, 39.4% of professional athletes reported that performance anxiety was an inhibiting factor preventing them from reaching higher levels (Birrer and Morgan, 2010).

To cope with these challenges, professional athletes turn to advanced psychological coping strategies (Nicholls and Polman, 2007). The most commonly employed methods include problem-focused strategies, imagery techniques, positive self-talk, relaxation exercises, and seeking social support (Gould et al., 2002). High levels of perceived social support have been shown to be associated with lower levels of anxiety and stress symptoms among athletes (Rees and Freeman, 2010). Moreover, mental training techniques such as positive self-talk and imagery have been reported to facilitate athletes’ reappraisal of anxiety-inducing situations, enabling them to perceive such situations as challenges rather than threats (Hanton et al., 2008). In this regard, effective coping with sport anxiety supports not only the sustainability of performance but also the development of psychological resilience.

### 1.5 The present study

It is well established that when professional athletes experience conditions of decent work, they demonstrate higher levels of work engagement. A decent work environment enhances employees’ job satisfaction and commitment, while the perception of decent work has a significant positive effect on engagement (BowenXue et al., 2024). Research focusing on athletes has revealed that throughout their careers they encounter various adversities, including child labor, violence, doping, early specialization, overtraining, commercial pressures, weakening of social bonds, and exposure to pressure and mobbing through unions (Buy, 2024; Kukathas, 2020; Lennon, 2024; Shevchenko, 2013). These findings highlight the necessity of addressing professional athletes’ fundamental rights, such as human rights, job security, social protection, retirement, and health benefits, within the context of their working environments.

Accordingly, the present study investigates the indirect pathways linking decent work to work engagement among athletes through visions about future and sport anxiety. It is hypothesized that decent work predicts athletes’ levels of hope, optimism, and pessimism regarding their future career experiences, and that visions about future in turn shape their sport anxiety (Mortensen et al., 2013; Sääksi et al., 2023). Experiencing and perceiving decent and stable working conditions may foster a positive visions about future among athletes (Ronkainen and Ryba, 2018). In supportive environments where basic needs are met and rights are protected, athletes are more likely to remain hopeful and optimistic about their careers (Wan and Duffy, 2022). It is anticipated that hopeful and optimistic athletes supported by decent work will experience lower levels of sport anxiety. Athletes with a positive visions about future are more likely to approach competitions by focusing on the possibility of success rather than fearing failure (Gordon, 2008; Lipowski, 2012). In other words, optimism is associated with lower anticipatory anxiety and a more constructive motivational stance. Empirical evidence corroborates the negative relationship between optimism and competitive anxiety, indicating that more optimistic athletes report significantly lower pre-competition anxiety and demonstrate higher performance (Ortín-Montero et al., 2017; Wilson et al., 2002). Therefore, if decent work fosters optimism and reduces pessimism among athletes, it may help alleviate sport anxiety (Wilson et al., 2002). Ultimately, this process can contribute to enhancing professional athletes’ work engagement.

On the other hand, in the Turkish context, where professional athletes often face unique career challenges such as education, contractual uncertainties, and inadequate social support, the decent work perspective is particularly relevant (FitGit Blog, 2023; Öztürk, 2022; Urhan and Fişne, 2022). Several key factors make the Turkish context noteworthy. Athletes encounter multidimensional challenges at every stage of their careers, including education, active competition, and the post-athletic period (Koçak et al., 2023; Yaman-Yılmaz, 2021). Insufficient support in education and dual career development undermines athletes’ personal growth and future planning (Ataçocuğu and Zelyurt, 2016; Yaman-Yılmaz, 2021); contractual uncertainties and precarious working conditions (Baştuğ, 2022; Ötkan and Çolakoğlu, 2025) jeopardize their economic and professional rights; while lack of social support and weak unionization leave athletes vulnerable both psychologically (Madan and Yoruç Çotuk, 2025) and legally (Ötkan and Çolakoğlu, 2025). The decent work perspective, by its very structure, offers a framework for athletes to experience a dignified and sustainable career (ILO, 2002). When considered in the Turkish context, this perspective reveals the need for improvements at every stage—from education to working conditions to post-retirement. Therefore, it is suggested that adopting the decent work perspective among professional athletes can provide significant contributions in addressing both individual and work-related dimensions within the Turkish context.

The career span of professional athletes is typically short, and their job security is low (Baştuğ, 2020). Even successful athletes may experience uncertainty regarding the continuity of their profession throughout their careers (Tunçkol, 2007; U.S. Bureau of Labor Statistics, 2024). Indeed, research has shown that many professional athletes work without contracts, fail to receive payments such as transfer fees and bonuses on time, lack retirement rights, and remain without adequate insurance coverage against injuries despite the high health risks inherent in sports (Union, 2013). Furthermore, it has been emphasized that many professional athletes are not legally recognized as “employees” and are therefore deprived of basic rights such as retirement pensions or maternity leave (O’Leary et al., 2025). A report published by the International Labour Organization (2020) also indicated that athletes are often not independently represented and, due to forms of hidden employment, cannot benefit from ordinary labor rights. The very nature of the sports industry, which expects athletes to be constantly available, negatively impacts work–life balance; constant work pressure leads to burnout and high turnover rates (Graham and Smith, 2022). Taken together, these findings suggest that our study, by quantitatively addressing the concept of decent work in the context of professional athletes, provides a valuable perspective to the sports literature and holds potential to make visible contributions to addressing job insecurity and deficits in social rights within the sports industry. Moreover, the study aims to offer findings that can guide researchers in sport management and human resources, as well as club and federation managers, athlete unions, and policymakers in establishing positive goals for athletes’ career pathways.

Building on this perspective, the present study emphasizes the significance of the decent work paradigm in professional sports. According to the existing literature, our study is among the pioneering efforts to quantitatively assess perceptions of decent work within a sample of professional athletes. Previous research on decent work has predominantly focused on traditional occupations such as healthcare, education, and service sectors (Bayrak, 2018; BowenXue et al., 2024; Gültaç, 2023; Saruhan, 2017; Yıldırımalp and İslamoğlu, 2014). By contrast, this study seeks to extend the concept of decent work into the realm of professional sports, aiming to elucidate its place within the field of sport sciences.

Ensuring fair, safe, and supportive working conditions—fundamental components of decent work—has been shown to protect professional athletes against adverse outcomes such as excessive stress and burnout, while simultaneously enhancing their work engagement (International Labour Organization [ILO], 1999; Su and Chan, 2023). Research indicates that individuals who feel secure, valued, and involved in their work are more focused and energetic, which in turn leads to improved performance outcomes (Christian et al., 2014; Yao et al., 2022). Conversely, excessive competitive anxiety and related psychological stressors have been shown to undermine athletic performance, particularly under high-pressure conditions (Woodman and Hardy, 2003). These findings underscore the importance of addressing mental health among professional athletes (Werner et al., 2023). Indeed, studies reveal that professional athletes experience mental health problems such as burnout, depression, and anxiety, which can directly impair their performance. Achieving peak performance therefore requires giving equal priority to athletes’ mental health alongside their physical preparation (American College of Sports Medicine, 2021; Reardon et al., 2019; Werner et al., 2023).

**Figure 1:**
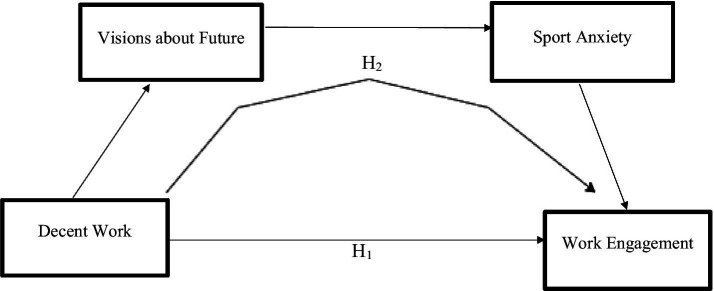
Hypothetical serial mediation model.

In summary, the present study emphasizes that decent work is an applicable factor in sports, closely linked to athletes’ psychological health. Furthermore, by demonstrating that concepts originating from organizational behavior (e.g., work engagement and decent work) can be meaningfully examined within the sport context, this study aims to contribute to bridging the gap between sport science and work psychology. Accordingly, the purpose of this research is to investigate the serial mediating role of visions about future (hope, optimism, and pessimism) and sport anxiety in the relationship between decent work and work engagement. In line with this purpose, the following hypotheses have been developed (Figure 1).

*H_1_*: Decent work is positively related to work engagement.

*H_2_*: The relationship between decent work and work engagement is serially mediated by visions about future (hope, optimism, and pessimism) and sport anxiety.

## 2 Materials and methods

### 2.1 Participants

The participants of the study are professional athletes who are active in professional football, volleyball and basketball leagues in Türkiye. Data from 296 professional athletes were used in the study. 89 (30.1%) of the participants were female and 207 (69.9%) were male. Participants’ ages ranged from 18 to 48, with an average age of 25.3 (SD = 6.49). The distribution of participants by branches is as follows: 163 football (55.1%), 102 volleyball (34.5%), 31 basketball (10.5%). The number of years that the participants have been working professionally varies between 1 and 32, and the average number of years that the participants have been working professionally is 8.29 (SD = 5.03).

### 2.2 Measurements

#### 2.2.1 Decent work scale

It was developed by Duffy et al. (2017) to measure the decent work perceptions of individuals working. It was adapted into Turkish by Buyukgoze-Kavas and Autin (2019). It consists of 5 sub-dimensions and 15 items. The subscales are as follows: “Physically and interpersonally safe working conditions,” “Access to healthcare,” “Adequate compensation,” “Hours that allow for free time and rest,” “Organizational values complement family and social values.” It is a 7-point Likert type (1: Strongly disagree to 7: Strongly agree). In this current study, the Cronbach alpha internal consistency coefficient calculated for the total score is 0.77.

#### 2.2.2 Utrecht work engagement scale

It was developed by Schaufeli et al. (2002) to determine the work engagement levels of individuals. Its adaptation into Turkish was carried out by Eryılmaz and Doğan (2012). It consists of three sub-dimensions and 17 items. The sub-dimensions are vigor, dedication and absorption. The scale is a 5-point Likert type (1: Not at all appropriate to 5: Completely appropriate). In this current study, the Cronbach alpha internal consistency coefficient calculated for the total score is 0.92.

#### 2.2.3 Visions about future scale

It was developed by Ginevra et al. (2017) to reveal the levels of hope, optimism and pessimism of individuals regarding the future as a whole. Its adaptation to Turkish was carried out on high school students by Akça et al. (2018). Construct validity was examined by Korkmaz (2023) on university students aged 17–27. The goodness of fit indices obtained for the confirmatory factor analysis are as follows: *χ*^2^ = 419.785, df = 132, *χ*^2^/df = 3.18, *p* < 0.001; GFI = 0.903; CFI = 0.914, RMSEA = 0.07 (LO = 0.06, HI = 0.08). The goodness of fit values of the confirmatory factor analysis conducted with data obtained from professional athletes within the scope of this current study are as follows: *χ*^2^ = 346.667, df = 121, *χ*^2^/df = 2.87, *p < 0*.001, GFI = 0.914, CFI = 0.922, RMSEA = 0.080 (LO90 = 0.070, HI90 = 0.089). The Turkish form of the scale consists of three sub-dimensions and 18 items. The sub-dimensions are hope, optimism and pessimism and the scale is a 5-point Likert type (1: Does not describe me at all to 5: Describes me very well). In this current study, Cronbach alpha internal consistency coefficients were calculated as 0.87 for hope, 0.79 for optimism, and 0.72 for pessimism.

#### 2.2.4 Sport anxiety scale

It was developed by Smith et al. (1990) to determine the sports anxiety levels of athletes. It was later revised by Smith et al. (2006). It was adapted into Turkish by Karadağ and Aşçı (2020) on adolescent athletes. The scale consists of three sub-dimensions and 15 items. Sub-dimensions are somatic anxiety, worry, concentration disruption. It is a 4-point Likert type (1: Not at all to 4: A lot). In this current study, the goodness of fit values of the second-order confirmatory factor analysis performed with data obtained from professional athletes are as follows: *χ*^2^ = 226.587, df = 81, *χ*^2^/df = 2.80, *p* < 0.001, GFI = 0.914, CFI = 0.913, RMSEA = 0.078 (LO90 = 0.066, HI90 = 0.090). The Cronbach alpha internal consistency coefficient calculated for the total score of the scale from the data obtained within the scope of this current study is 0.83.

### 2.3 Procedure

All procedures of the current study were carried out in accordance with the 1975 Helsinki Declaration. Before starting to collect the data, ethical approval was obtained from the Karamanoğlu Mehmetbey University Social and Human Sciences Scientific Research and Publication Ethics Committee of the institution to which the authors are affiliated (Date and number: 18.02.2025–245,115). Data was collected through an online tool (Google Forms). During the data collection process, communication officers of the sports clubs were contacted, and the data collection instruments were distributed to the athletes via their communication groups. Informed consent was obtained from participants before data collection. All data used in the study were obtained from voluntary participants.

### 2.4 Data analysis

A total of 380 professional athletes were reached in the research. Data belonging to 69 people who did not give the expected response to all of the two validity items placed among the measurement tools (i.e., mark this item as 3) were excluded from the analysis on the grounds that the scale battery was not filled in carefully. Before the analyses, extreme values regarding the scores obtained from the scales were examined. *Z* scores outside +/− 3 were considered extreme values and were removed from the data set (Tabachnick and Fidell, 2019). Data from a total of 15 individuals were excluded from the analysis: two from work engagement, five from sports anxiety, and eight from pessimism. In the last case, the analyses were performed on a dataset of 296 professional athletes.

When the kurtosis and skewness values of the scores used in the study are examined, it is seen that all values are between +1.5 and −1.5 (Table 1). Accordingly, it can be said that the distributions of the variables used in the study are normal (Tabachnick and Fidell, 2019). Descriptive statistics of the variables were calculated and the relationships between the variables were obtained by calculating the Pearson Correlation coefficient (*r*). Structural equation modeling was used to test the hypothetical model of the research. To test the significance of the model, the following criteria were taken into account: *χ*^2^, *χ*^2^/df ≤ 5, RMSEA ≤ 0.10, GFI, CFI ≥ 0.90 (Hu and Bentler, 1999; Tabachnick and Fidell, 2019). In order to test the significance of the mediation effects, the bootstrapping method (5,000 reiterations) was preferred. For the significance of direct and indirect effects, confidence intervals should not include zero (Preacher and Hayes, 2008). IBM SPSS Statistics 29 and IBM SPSS AMOS 27 programs were used in the analysis of the data.

**Table 1:** Descriptive statistics and correlation coefficients.

Variables	*x̄*	SD	1	2	3	4	5	6
1. Decent work	71.21	13.80		0.32^***^	0.29^***^	−0.31^***^	−0.06	0.44^***^
2. Hope	30.39	4.31			0.56^***^	−0.54^***^	−0.29^***^	0.49^***^
3. Optimism	24.84	3.57				−0.45^***^	−0.33^***^	0.38^***^
4. Pessimism	8.29	2.98					0.30^***^	−0.44^***^
5. Sport anxiety	21.23	5.31						−0.46^***^
6. Work engagement	74.56	9.90						
Skewness			−0.08	−0.82	−0.70	0.51	1.22	−1.16
Kurtosis			−0.28	0.38	0.51	−0.62	1.17	0.99

***p < 0.001. *N* = 296.

## 3 Results

The descriptive statistics and correlation coefficients are presented in Table 1.

When Table 1 is examined, it is seen that there is a positive relationship between decent work and work engagement (*r* = 0.44, *p* < 0.001), hope (*r* = 0.32, *p* < 0.001) and optimism (*r* = 0.29, *p* < 0.001). On the other hand, while there is a negative relationship between decent work and pessimism (*r* = −0.31, *p* < 0.001), there is no significant relationship between sports anxiety. In addition, there are negative relationships between work engagement and sport anxiety (*r* = −0.46, *p* < 0.001) and pessimism (*r* = −0.44, *p* < 0.001). On the other hand, there are positive relationships between work engagement and optimism (*r* = 0.38, *p* < 0.001) and hope (*r* = 0.49, *p* < 0.001).

### 3.1 The serial mediation analysis findings

**Figure 2:**
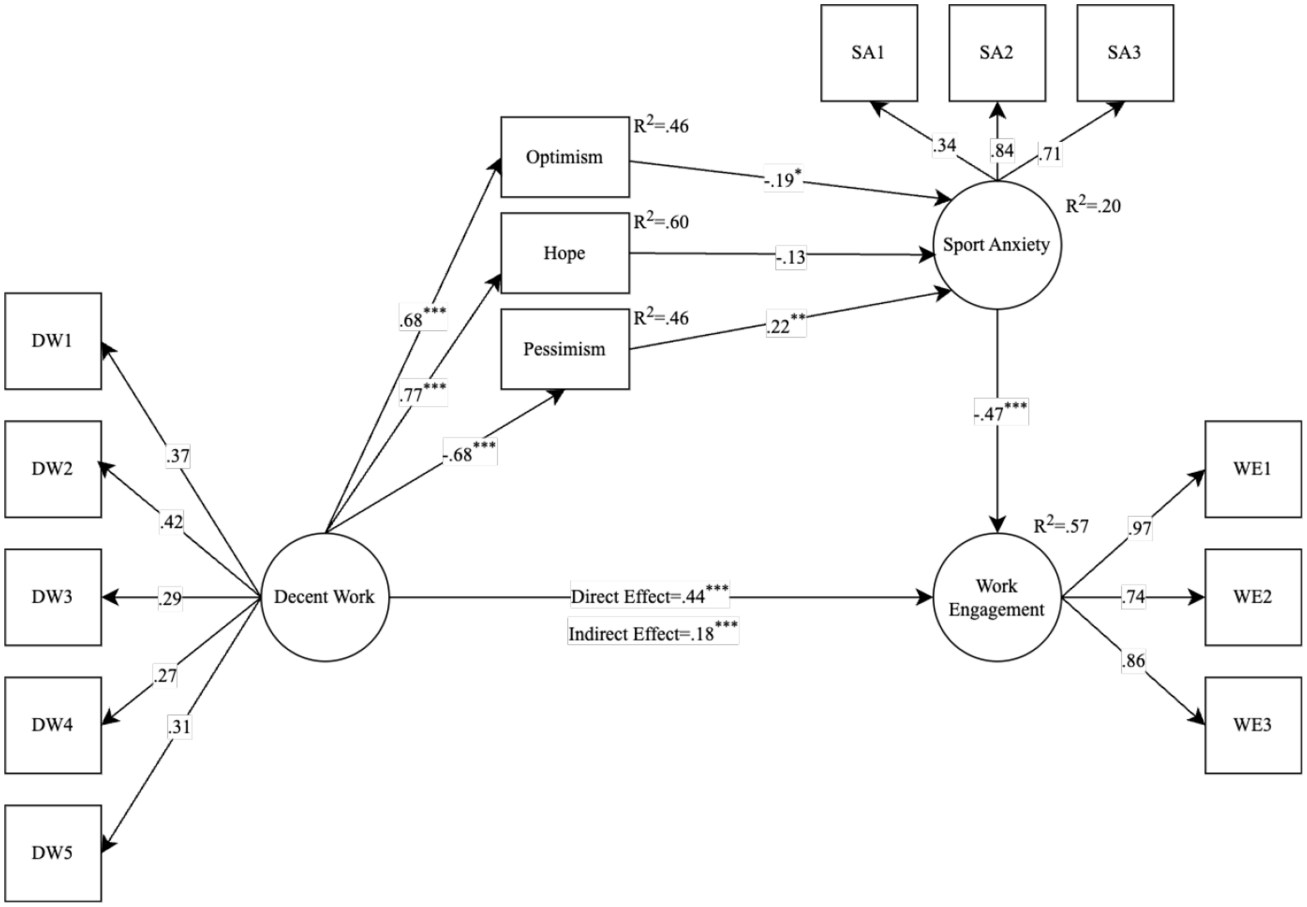
Findings of the hypothetical serial mediation model. ^*^p < 0.05, ^**^p < 0.01, ^***^p < 0.001.

In serial mediation analysis, the mediating role of visions about future (hope, optimism, pessimism) and sport anxiety in the relationship between decent work and work engagement was examined. The findings regarding the research model are presented in Figure 2.

Goodness of fit values for the model were obtained above the accepted criteria: *χ*^2^ = 194.392, df = 68, *χ*^2^/df = 2.86, *p* < 0.001, GFI = 0.910, CFI = 0.915, RMSEA = 0.079 (LO90 = 0.066, HI90 = 0.093). When Figure 2 is examined, it is seen that decent work together with the mediating variables explain 57% of the variance of work engagement (*R*^2^ = 0.57). When the direct effects are examined, it is seen that the direct effect of decent work on optimism (*β* = 0.68, *p* < 0.001), hope (*β* = 0.77, *p* < 0.001) and work engagement (*β* = 0.44, *p* < 0.001) is positive and significant. On the other hand, the direct effect of decent work on pessimism is negative and significant (*β* = −0.68, *p* < 0.001). In addition, the direct effect of optimism on sport anxiety is negative and significant (*β* = −0.19, *p* < 0.05), while the direct effect of pessimism on sport anxiety is positive and significant (*β* = 0.22, *p* < 0.01). The direct effect of hope on sport anxiety is not significant. Finally, the direct effect of sport anxiety on work engagement is negative and significant (*β* = −0.47, *p* < 0.001). The indirect effect of decent work via visions about future (optimism, pessimism) and sport anxiety is significant. In other words, it was observed that visions about future (optimism, pessimism) and sport anxiety played a significant serial mediation role in the relationship between decent work and work engagement. The only path where serial mediation is not significant is the hope path. Bootstrap intervals obtained on other paths except hope did not include zero. This shows the serial mediation role of visions about future (optimism, pessimism) and sport anxiety. [*β* = 0.18, BC-Bias 95% lower-bound = 0.12, upper-bound = 0.25]. The findings of the serial mediation analysis are presented in Table 2.

**Table 2:** Bootstrapping findings of the hypothetical serial mediation model.

Pathway	*B*	S. E.	C. R.	Coefficient	Lower bound	Upper bound
Direct effects						
DW → Optimism	2.41	0.76	3.19	0.68^***^	0.58	0.77
DW → Hope	3.32	1.03	3.22	0.78^***^	0.71	0.83
DW → Pessimism	−1.99	0.63	−3.19	−0.68^***^	−0.75	−0.59
Optimism → SA	−0.03	0.01	−2.36	−0.19^*^	−0.33	−0.05
Hope → SA	−0.02	0.01	−0.67	−0.13	−0.28	0.03
Pessimism → SA	0.05	0.02	2.66	0.22^**^	0.12	0.33
SA → WE	1.59	0.53	3.02	−0.47^***^	−0.59	−0.34
Total indirect effect						
DW → VAF → SA → WE				0.18^***^	0.12	0.25
Indirect effects						
DW → SA				−0.38^***^	−0.46	−0.29
Optimism → SA → WE				0.09^*^	0.03	0.15
Hope → SA → WE				0.06	−0.007	0.15
Pessimism → SA → WE				−0.10^***^	−0.18	−0.05
Total effect						
DW → WE				0.62^***^	0.55	0.68

*N* = 296. ^*^p < 0.05, ^**^p < 0.01, ^***^p < 0.001. DW, decent work; SA, sport anxiety; WE, work engagement; VAF, visions about future.

## 4 Discussion

The findings of the current study demonstrate that professional athletes’ perceptions of decent work substantially influence their work engagement through future-oriented cognitions and emotional responses. In particular, perceiving work as decent was associated with higher levels of hope and optimism and markedly lower levels of pessimism. Optimism and pessimism, in turn, were linked to reduced sport anxiety, which subsequently enhanced work engagement. These results highlight the protective role of optimism in alleviating sport anxiety, while pessimism emerged as a risk factor that exacerbates it. Elevated sport anxiety was found to significantly undermine athletes’ work engagement. Notably, the effect of hope on sport anxiety did not reach statistical significance. Moreover, the serial mediation analysis indicated that the relationship between decent work and work engagement operates through optimism and pessimism, along with sport anxiety: optimism and pessimism jointly serve as meaningful serial mediators between decent work and work engagement, whereas hope does not. Overall, the findings suggest that perceiving work as decent strengthens positive visions about future, mitigates sport anxiety, and ultimately enhances athletes’ work engagement.

Our study demonstrates that athletes’ perceptions of decent work significantly strengthen their positive visions about future. Athletes who perceive their work as decent report higher levels of optimism and hope, accompanied by a notable reduction in pessimism. This result aligns with prior research suggesting that decent work fosters employees’ psychological capital. For example, Ferraro et al. (2018), in their study with knowledge workers in Portugal and Brazil, showed that perceptions of decent work meaningfully enhanced psychological capital, including optimism and hope as essential components of future-oriented beliefs. Bandura’s (2013) social cognitive perspective further posits that positive reinforcement and support within the work environment can consolidate individuals’ beliefs about the future. Consistently, the Psychology of Working Theory (Duffy et al., 2016) identifies decent work as a pivotal determinant that fulfills employees’ basic needs while simultaneously promoting well-being and positive expectations about the future. Taken together, these findings underscore that professional athletes’ perceptions of decent work constitute a crucial factor in fostering optimism and hope, thereby strengthening their positive future orientations.

Another key result of the study is the significant link between athletes’ visions about future, specifically optimism and pessimism, and their levels of sport anxiety. Athletes with higher levels of optimism reported substantially lower performance anxiety, whereas those characterized by stronger pessimistic tendencies exhibited elevated anxiety levels. These findings are consistent with Wilson et al. (2002), who demonstrated that optimistic college athletes experienced markedly lower pre-competition anxiety compared to both “defensive pessimists” and “real pessimists,” while athletes relying on pessimistic strategies reported generally higher anxiety levels. Similarly, Orhan et al. (2024) emphasized that optimism functions as a psychological resource that promotes adaptive coping strategies and resilience, thereby facilitating high performance. In contrast, pessimism contributes to maladaptive responses and mental health difficulties, ultimately impairing athletic performance. Furthermore, research has consistently shown that optimism is a positive predictor of sport performance (Ortín-Montero et al., 2017). Collectively, these findings highlight optimism as a protective factor against sport anxiety, while pessimism emerges as a vulnerability factor that undermines athletic functioning.

The findings of the present study further revealed that hope did not exert a significant effect on sport anxiety. This outcome is consistent with theoretical perspectives on the role of hope. According to Snyder et al. (2002), hope represents a cognitive framework encompassing both pathways (planning) and agency (motivation) required to achieve goals. Thus, hopeful individuals are characterized by strong motivation toward their future objectives and the capacity to identify alternative routes for attaining them. However, because hope primarily reflects a goal-directed belief in personal effort, it may not directly alter athletes’ perceptions of immediate risks and threats in competitive environments. Conceptually, this distinction resonates with Bryant and Cvengros (2004), who argued that optimism reflects a generalized expectation of positive outcomes, whereas hope emphasizes confidence in achieving goals through personal agency. From this perspective, athletes’ hopefulness may orient them toward long-term career goals, yet it may not buffer anxiety in the context of imminent competitions. In sum, while optimism generates a more “calming” expectancy that alleviates anxiety, hope operates as a ‘goal-oriented’ motivational force, and this may explain why it was not a significant predictor of sport anxiety in this study.

Another important result of this study is that sport anxiety exerts a detrimental effect on professional athletes’ work engagement. Athletes who experience elevated levels of competitive anxiety may struggle to fully commit to their work, that is, to their sport and team. This often results in reduced motivation and heightened emotional exhaustion. This pattern is consistent with broader evidence in the work psychology literature, where numerous studies have identified work-related anxiety and stress as key factors undermining employees’ engagement and motivation. For instance, Ashford et al. (1989) found that anxiety stemming from job insecurity diminished employees’ organizational commitment and job satisfaction. Similarly, Thu et al. (2024) reported that workplace anxiety reduces work engagement and increases the likelihood of employee withdrawal. Moreover, longitudinal evidence highlights the protective role of work engagement for psychological well-being. A two-year study conducted in Norway across eight occupational groups revealed that employees with higher levels of work engagement reported significantly lower anxiety symptoms 2 years later (Innstrand et al., 2012). Taken together, these findings corroborate the negative association between anxiety and work engagement.

The serial mediation model tested in this study demonstrated that optimism, pessimism, and sport anxiety sequentially mediate the relationship between decent work and work engagement. Specifically, perceptions of decent work shape athletes’ visions about future, including levels of hope, optimism, and pessimism. When athletes perceive greater value and fairness in their work environment, they tend to display higher optimism and reduced pessimism. These cognitive orientations subsequently influence levels of sport anxiety. Consistent with Lazarus’s appraisal framework, increases in optimism reduce perceived threats and thereby decrease anxiety, whereas heightened pessimism elevates stress and exacerbates anxiety (Krohne, 2001). At the final stage of the process, lower sport anxiety facilitates greater work engagement among athletes. Thus, decent work positively contributes to work engagement through the pathway of enhanced optimism and reduced sport anxiety. Conversely, limited perceptions of decent work foster pessimism and heightened anxiety, which in turn undermine engagement. In sum, the findings highlight a meaningful sequential mediation mechanism linking decent work to work engagement, operating through the dual pathways of “optimism leading to low anxiety” and “pessimism leading to high anxiety.

### 4.1 Conclusion

In conclusion, this study demonstrates that professional athletes’ perceptions of decent work play a central role in fostering work engagement by shaping their visions about future and regulating sport anxiety. Specifically, perceiving work as decent enhances optimism and reduces pessimism, which in turn lowers sport anxiety and strengthens athletes’ engagement in their professional commitments. Although hope did not emerge as a significant mediator, the sequential pathways of “optimism → low sport anxiety” and “pessimism → high sport anxiety” provide important insights into the mechanisms through which decent work influences athletes’ psychological functioning and career sustainability. Overall, these findings underscore the value of promoting decent work conditions within professional sports, not only to improve athletes’ immediate well-being and motivation but also to support their long-term engagement, resilience, and sustainable career development.

### 4.2 Limitations and future research

For future research, the findings of this study can be extended in several directions. First, the causal structure of the sequential mediation model should be examined through longitudinal and experimental designs, enabling the effects of decent work interventions to be tracked over time. Second, similar models could be tested among amateur athletes in Türkiye to explore their broader applicability within non-professional sport settings. In addition, the model could be examined in sport branches such as wrestling and athletics, which differ in audience profile and economic structure yet share comparable developmental characteristics. Furthermore, the model could be tested in other developing countries that fall within the same income group as Türkiye, allowing for broader evaluations of its validity and generalizability across comparable socio-economic contexts. In particular, future research may investigate whether the mechanisms operate differently in individual versus team sports or between male and female athletes. Third, additional mediating and moderating variables such as psychological resilience, self-efficacy, and social support could be incorporated into the model to uncover other processes influencing athletes’ work engagement. Fourth, future studies should consider the limitations of relying exclusively on self-report measures, as such designs may be subject to social desirability bias and shared method variance. Incorporating multi-method approaches, such as coach evaluations or physiological indicators of anxiety, could strengthen the robustness of the findings. Finally, examining the impact of decent work on athletes’ objective performance indicators (e.g., competition results or statistical records) would provide valuable insights into the relationship between psychosocial well-being and concrete outcomes in sport. Moreover, the current study was conducted with athletes in Türkiye, and future research should replicate and extend these findings in broader and more diverse samples to enhance cross-cultural validity.

The findings of this study offer several actionable insights for practice. Promoting fair, safe, and stable working conditions may enhance athletes’ optimism, reduce anxiety, and strengthen their engagement. Coaches and sport managers can design training environments that support psychological security and incorporate optimism-based mental training to help athletes cope with pressure more effectively. Federations and policymakers, particularly in Türkiye and other developing countries with similar socio-economic conditions, could integrate the decent work framework into sport governance and employment policies to foster athletes’ well-being, motivation, and sustainable career development.

## Data Availability

The raw data supporting the conclusions of this article will be made available by the authors, without undue reservation.
